# Correction: Design and Selection of a Camelid Single-Chain Antibody Yeast Two-Hybrid Library Produced *De Novo* for the Cap Protein of Porcine Circovirus Type 2 (PCV2)

**DOI:** 10.1371/journal.pone.0196619

**Published:** 2018-04-24

**Authors:** The PLOS ONE Staff

## Notice of republication

This article was republished on March 28, 2018 to correct for the inadvertent use of improper language in the caption of Figure 8. The publisher apologizes for the lack of oversight and any offense caused. Please download this article again to view the correct version.
